# Sodium-glucose cotransporter 2 inhibitor may not prevent atrial fibrillation in patients with heart failure: a systematic review

**DOI:** 10.1186/s12933-023-01860-1

**Published:** 2023-05-24

**Authors:** Xiaolan Ouyang, Jiafu Wang, Qian Chen, Long Peng, Suhua Li, Xixiang Tang

**Affiliations:** 1 Department of Cardiovascular Medicine, the Third Affiliated Hospital, Sun Yat-sen University, Guangzhou, China; 2 VIP medical service center, the Third Affiliated Hospital, Sun Yat-sen University, Guangzhou, China

**Keywords:** Sodium-glucose cotransporter 2 inhibitor, Atrial fibrillation, Heart failure

## Abstract

**Background:**

Atrial fibrillation (AF) and heart failure (HF) frequently coexist because of their similar pathological basis. However, whether sodium-glucose cotransporter 2 inhibitor (SGLT2i), a novel class of anti-HF medication, decreases the risk of AF in HF patients remains unclear.

**Objectives:**

The aim of this study was to assess the relationship between SGLT2i and AF in HF patients.

**Methods:**

A meta-analysis of randomized controlled trails evaluating the effects of SGLT2i on AF in HF patients was performed. PubMed and ClinicalTrails.gov were searched for eligible studies until 27 November 2022. The risk of bias and quality of evidence were assessed through the Cochrane tool. Pooled risk ratio of AF for SGLT2i versus placebo in eligible studies was calculated.

**Results:**

A total of 10 eligible RCTs examining 16,579 patients were included in the analysis. AF events occurred in 4.20% (348/8292) patients treated with SGLT2i, and in 4.57% (379/8287) patients treated with placebo. Meta-analysis showed that SGLT2i did not significantly reduce the risk of AF (RR 0.92; 95% CI 0.80–1.06; *p* = 0.23) in HF patients when compared to placebo. Similar results remained in the subgroup analyses, regardless of the type of SGLT2i, the type of HF, and the duration of follow-up.

**Conclusions:**

Current evidences showed that SGLT2i may have no preventive effects on the risk of AF in patients with HF.

**Translational perspective:**

Despite HF being one of the most common heart diseases and conferring increased risk for AF, affective prevention of AF in HF patients is still unresolved. The present meta-analysis demonstrated that SGLT2i may have no preventive effects on reducing AF in patients with HF. How to effectively prevent and early detect the occurrence of AF is worth discussing.

**Supplementary Information:**

The online version contains supplementary material available at 10.1186/s12933-023-01860-1.

## Introduction

Atrial fibrillation (AF) frequently coexists with heart failure (HF), and increases the risk of worse events and complexity of treatment [1, 2]. AF in patients with HF has a more intricate pathological mechanism, whereby HF contributes to the electrical and structural remodeling of the heart, and promotes vulnerability to the development of AF [3–5]. Given the multitude of studies that have emphasized the increased risk associated with AF [6–11], it is worthwhile to prioritize early detection and novel treatment strategies for AF in patients with HF. Among current anti-HF drugs, renin-angiotensin system inhibitors [12], beta blockers [13], anti-mineralocorticoid [14, 15] and eplerenone have been proved to reduce the risk of new-onset AF (NOAF), whereas vericiguat [10] and spironolactone [16] seem to have little impact on the occurrence of AF. Sodium-glucose cotransporter 2 inhibitor (SGLT2i) have emerged as a promising first-line treatment option for HF, as it can effectively lower the risk of hospitalization and cardiovascular mortality in patients with HF. In addition, SGLT2i is believed to mitigate atrial fibrosis, myocardial hypertrophy and improves mitochondrial function [17–19], supporting the potential use of SGLT2i to reduce the risk of AF in HF patients. However, previous trials examining the effects of SGLT2i on the incidence of AF have reported conflicting results [20]. Therefore, the present meta-analysis aimed to summarize the relevant literatures to provide insights into the controversy over the association between SGLT2i and AF in HF patients with both reduced and preserved ejection fraction (HFrEF and HFpEF).

## Methods

### Data sources and search strategy

The present meta-analysis was conducted and reported according to the Preferred Reporting Items for Meta-Analyses (PRISMA) statement [21] (Supplemental Table 1). PubMed and Clinicaltrails.gov were searched until 27 November 2022. The following key words were used for search without further restrictions: (a) SGLT2i-related terms, including “empagliflozin”, “dapagliflozin”, “canagliflozin”, “ipragliflozin”, “ertugliflozin”, “sotagliflozin”, “luseogliflozin”, (b) heart failure, and (c) atrial fibrillation. The reference lists of retrieved articles were also scrutinized to identify additional relevant literatures. Books and documents, meta-analysis, review, and systematic review were excluded.

### Study selection

Eligibility criteria for included studies required (1) randomized controlled trails (RCTs); (2) participants with a confirmed diagnosis of HFrEF [left ventricular ejection fraction ≤ 40%] or HFpEF [left ventricular ejection fraction>40%]; (3) SGLT2i and placebo as the intervention; (4) adverse events/outcomes include AF. Excluded criteria mainly included (1) other positive drug interventions besides SGLT2i; (2) incomplete RCTs / RCTs without results reported. Publication year or language was not restricted.

### Outcome of interest

Primary outcome of interest is the incidence of AF events, which was collected and defined as AF reported in serious adverse event or other adverse events. Subgroup analyses focused on type of SGLT2i, duration of follow-up, and type of HF were conducted.

### Data extraction and quality assessment

The relevant information of each study included in the present analysis were retrieved, including the name of RCTs, the registration number, year of publication, name of SGLT2i, dosage of SGLT2i, sample size, mean age, mean follow-up duration, mean left ventricular ejection fraction (LVEF), gender, number of patients with type 2 diabetes, chronic kidney diseases and anti-HF drugs used. Unpublished data was obtaining form Clinicaltrails.gov database. The Cochrane Collaboration’s tool [22] was utilized for performing the quality assessment of the included studies. Every RCT contributing to the AF events was categorized as having low, high, or unclear quality according to seven domains: random-sequence generation (selection bias), allocation concealment (selection bias), blinding (performance bias and detection bias), incomplete outcome data (attrition bias), selective reporting (reporting bias) and other bias. Literature search, study selection, data extraction and quality assessment were carried out independently by two authors. Disagreement was resolved by consensus or by the corresponding author.

### Statistical analyses

Risk ratios (RRs) and 95% confidence intervals (CIs) were calculated for each study. After extracting the initial data, it was obvious that all qualified studies reported the same dosage of the use of SGLT2i. The percentage of variability across studies attributable to heterogeneity was estimated by using the Cochrane’s Q and I^2^; I^2^ < 50% was considered as low heterogeneity and I^2^ ≥ 50% as high heterogeneity. A fixed-effect model to combine results of the studies when I^2^ < 50%, while a random-effect model instead when I^2^ ≥ 50%. To assess the robustness of our finding, sensitivity and subgroup analyses were conducted: (1) estimates were recalculated after removing study one by one from the pooled analysis; (2) subgroup analyses were performed to assess the effect of limit conditions such as the type of SGLT2i, the type of HF and the follow-up time. Since all of the included RCTs employed the same dosage of SGLT2i, we did not carry out any further sub-analyses. The funnel plots [23] and Harbord test [24] were used to evaluate the possibility of publication bias. Results reached statistical significance when *p*<0.05. All operations were performed by using Review manager 5.3 and Stata software 15.0.

## Results

### Literature search

**Fig. 1:**
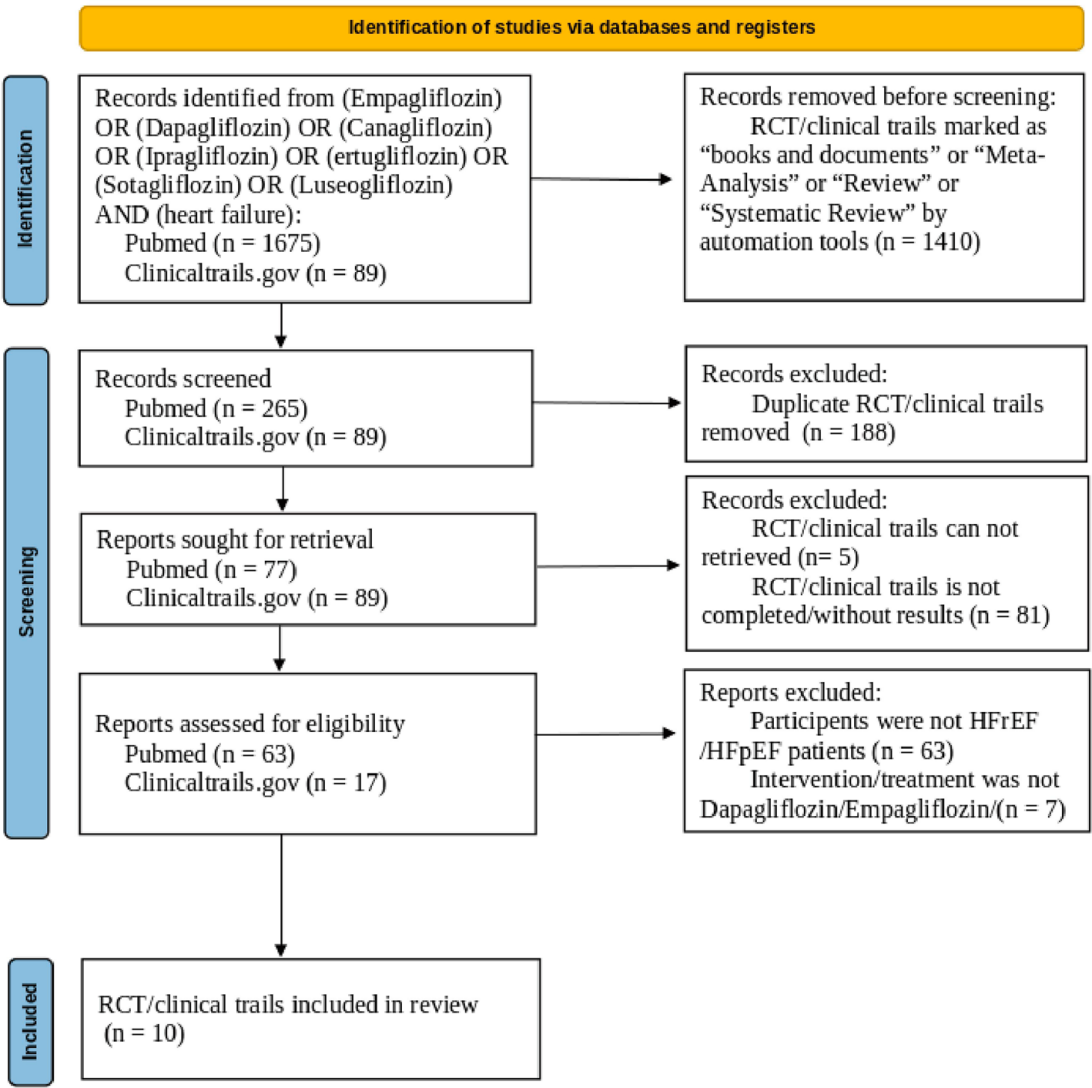
PRISMA flow diagram. HFpEF = heart failure with preserved ejection fraction; HFrEF = heart failure with reduced ejection fraction; RCT = randomized controlled trial

The flowchart illustrating study selection was shown in Fig. 1. Our searches yielded 77 records in PubMed and 89 records in Clinicaltrails.gov after rejecting 1,410 reports which were not marked as RCT/clinical trials and excluding 188 duplicated RCTs. Of the 166 studies sorted, 86 articles were not available for detailed data. After examining the full texts, 70 records that did not meet the inclusion criteria were removed. Finally, 10 unique eligible RCTs [9, 25–33] focusing on the comparison between SGLT2i and placebo were included for our analysis.

### Characteristics of included studies

Ten RCTs [9, 25–33] with 16,579 patients focused on the comparison between the use of SGLT2i and placebo. Of the 10 RCTs, 5 used dapagliflozin [9, 25–28] as the positive intervention, and 5 used empagliflozin [29–33]. The mean age ranged from 61.3 to 73.5 years and the mean follow-up time ranged from 12 weeks to 26.2 months. Baseline and key characteristics of the enrolled records were presented in Table 1.

**Table 1:** Baseline and main characteristics of the enrolled records

Study	Condition	Drug	Dose	Year	Number of participants(N)	LVEF (%), Mean	Follow-up time,Mean	Age(y), Mean	Male (%)	Diabetes (%)	eGFR < 60 ml/min/1.73 m (%)	ACEi/ARB/ARNi (N)	Beta-blocker (N)	MRA (N)
DAPA-HF	HFrEF	Dapagliflozin	10 mg	2019	4744	31.10%	18.2 months	66.3	76.60%	42.00%	41%	4459	4554	3368
DETERMINE-reduced	HFrEF	Dapagliflozin	10 mg	2021	313	NA	16 weeks	67.8	74.40%	NA	NA	NA	NA	NA
DETERMINE-preserved	HFpEF	Dapagliflozin	10 mg	2020	504	NA	16 weeks	71.8	63.50%	NA	NA	NA	NA	NA
DEFINE-HF	HFrEF	Dapagliflozin	10 mg	2019	263	26.40%	13 weeks	61.3	73.40%	63.10%	NA	241	256	160
PRESERVED-HF	HFpEF	Dapagliflozin	10 mg	2022	324	NA	12 weeks	70	43.20%	55.60%	NA	NA	NA	NA
EMPERIAL-Reduced	HFrEF	Empagliflozin	10 mg	2020	312	NA	12 weeks	69	74.40%	NA	NA	287	295	182
EMPERIAL-Preserved	HFpEF	Empagliflozin	10 mg	2020	315	NA	12 weeks	73.5	56.80%	NA	NA	246	281	105
EMPEROR-Reduced	HFrEF	Empagliflozin	10 mg	2020	3730	27.40%	16 months	66.8	76.10%	49.70%	48%	3327	3533	2661
EMPEROR-Preserved	HFpEF	Empagliflozin	10 mg	2020	5988	NA	26.2 months	71.8	55.30%	48.90%	24.88%	4839	5164	2240
SUGAR-DM-HF	HFrEF	Empagliflozin	10 mg	2022	205	32.50%	36 weeks	68.7	73.30%	78.10%	50.20%	NA	NA	NA

ACEi = angiotension converting enzyme inhibitors; ARB = angiotonin receptor blocker; ARNi = angiotensin receptor enkephalase inhibitors; eGFR = estimated glomerular filtration rate; HFpEF = heart failure with preserved ejection fraction; HFrEF = heart failure with reduced ejection fraction; LVEF = left ventricular ejection fraction; MRA = mineralocorticoid receptor antagonist; NA = not applicable; T2DM = type 2 diabetes;

Detailed results of the Cochrane risk of bias assessment are summarized in Supplemental Fig. 1. All included studies were described as randomized and double-blinded, and all records were registered on Clinicaltrails.gov and identified with a registration number. As AF event was reported as an adverse event rather than a primary or secondary outcome, bias may exist in reporting. Finally, all studies were assessed as being at low risk of bias (Supplemental Fig. 1).

### Impact of SGLT2i on AF in patients with HF

**Fig. 2:**
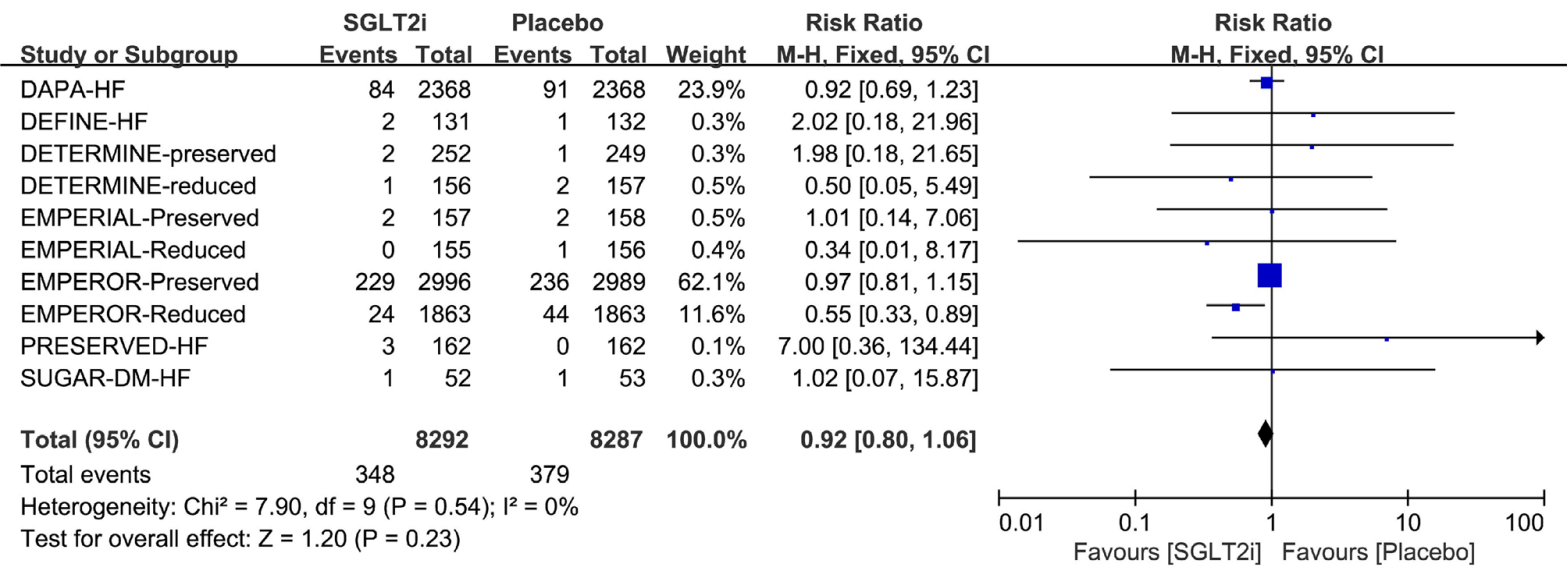
Forest plot of impact of SGLT2i on AF event in RCT. M-H = Mantel-Haenszel; AF = atrial fibrillation; SGLT2i = sodium-glucose cotransporter 2 inhibitor; RCT = randomized controlled trial

**Fig. 3:**
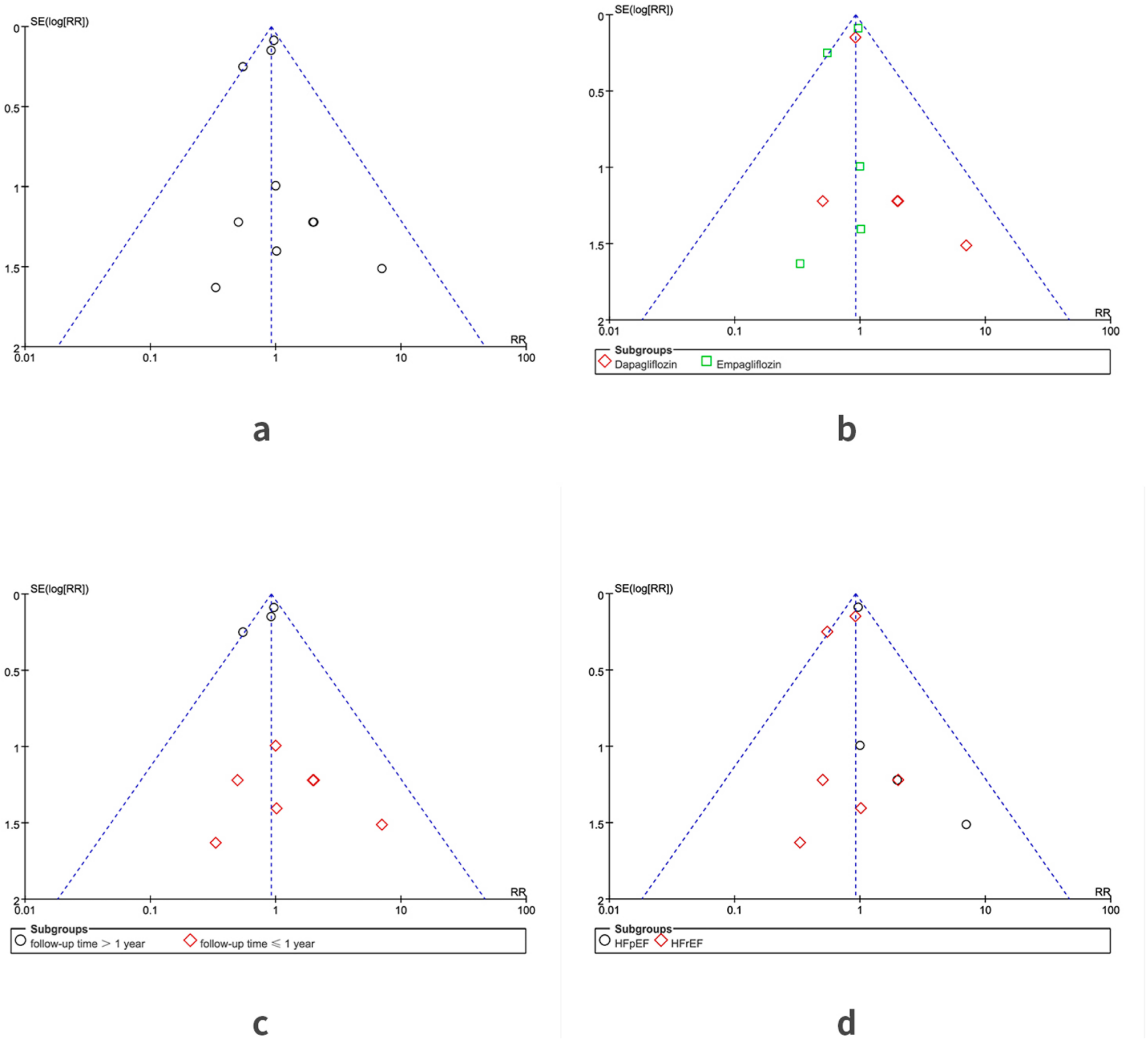
Funnel plot. (a) Funnel plot for assessing risk of bias appeared to be asymmetrical; (b) Funnel plot of subgroup analysis by drug use; (c) Funnel plot of subgroup analysis by follow-up time; (d) Funnel plot of subgroup analysis by type of HF. HF = heart failure; AF = atrial fibrillation; RCT = randomized controlled trial; RR = risk ratio; SGLT2i = sodium-glucose cotransporter 2 inhibitor

Of the 8292 patients treated with SGLT2i, 348 AF events were observed. While 379 AF events occurred among 8287 participants in the placebo group. The meta-analysis showed that SGLT2i did not significantly affect the risk of AF when compared with placebo (RR 0.92, 95%CI 0.80–1.06, *p* = 0.23) (Fig. 2). Additionally, no heterogeneity between trails was observed (*p* = 0.54; I^2^ = 0%) (Fig. 2). The funnel plot comparing the incidence of AF between SGLT2i and placebo, as shown in Fig. 3a, revealed no apparent asymmetry upon visual inspection. Moreover, the Harbord test did not show significant publication bias (*p* = 0.87; Supplemental Table 2).

### Subgroup analyses and sensitivity analyses

Outcomes of AF between SGLT2i and placebo kept unchanged after removing studies one by one from the analysis, as shown in Supplemental Fig. 2a.

**Fig. 4:**
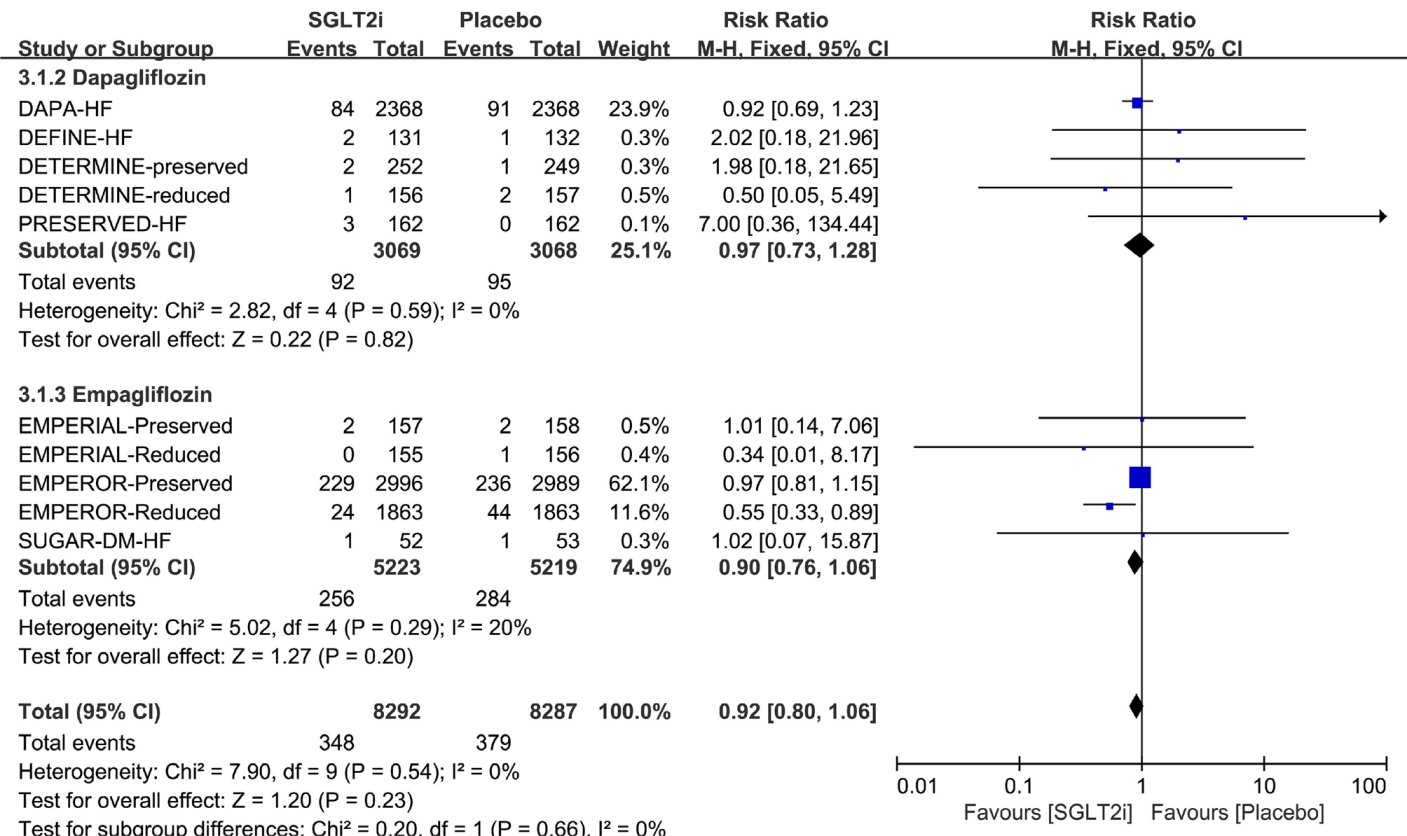
Forest plot of subgroup analysis by drug use. M-H = Mantel-Haenszel; SGLT2i = sodium-glucose cotransporter 2 inhibitor

The subgroup analysis based on the type of SGLT2i agent use revealed that neither dapagliflozin use [9, 25–28] (RR 0.97, 95% Cl 0.73–1.28, *p* = 0.82) nor empagliflozin use [29–33] (RR 0.90, 95% CI 0.76–1.06, *p* = 0.20) showed a significant reduction in the risk of AF (Fig. 4). The Harbord test indicated no publication bias for either dapagliflozin (*p* = 0.27) or empagliflozin use (*p* = 0.41) (Supplemental Table 2). As the subgroup limited to empagliflozin use performed low heterogeneity (*p* = 0.29, I^2^ = 20%), we further conducted sensitivity analysis by sequentially removing each study in empagliflozin group (Supplemental Fig. 2b). Interestingly, there was no heterogeneity existed after removing EMPEROR-preserved. Finally, in comparison with placebo, empagliflozin did reduced AF events (RR 0.57; 95% CI 0.36–0.91, *p* = 0.02; Supplemental Fig. 3) after sensitivity analyses.

**Fig. 5:**
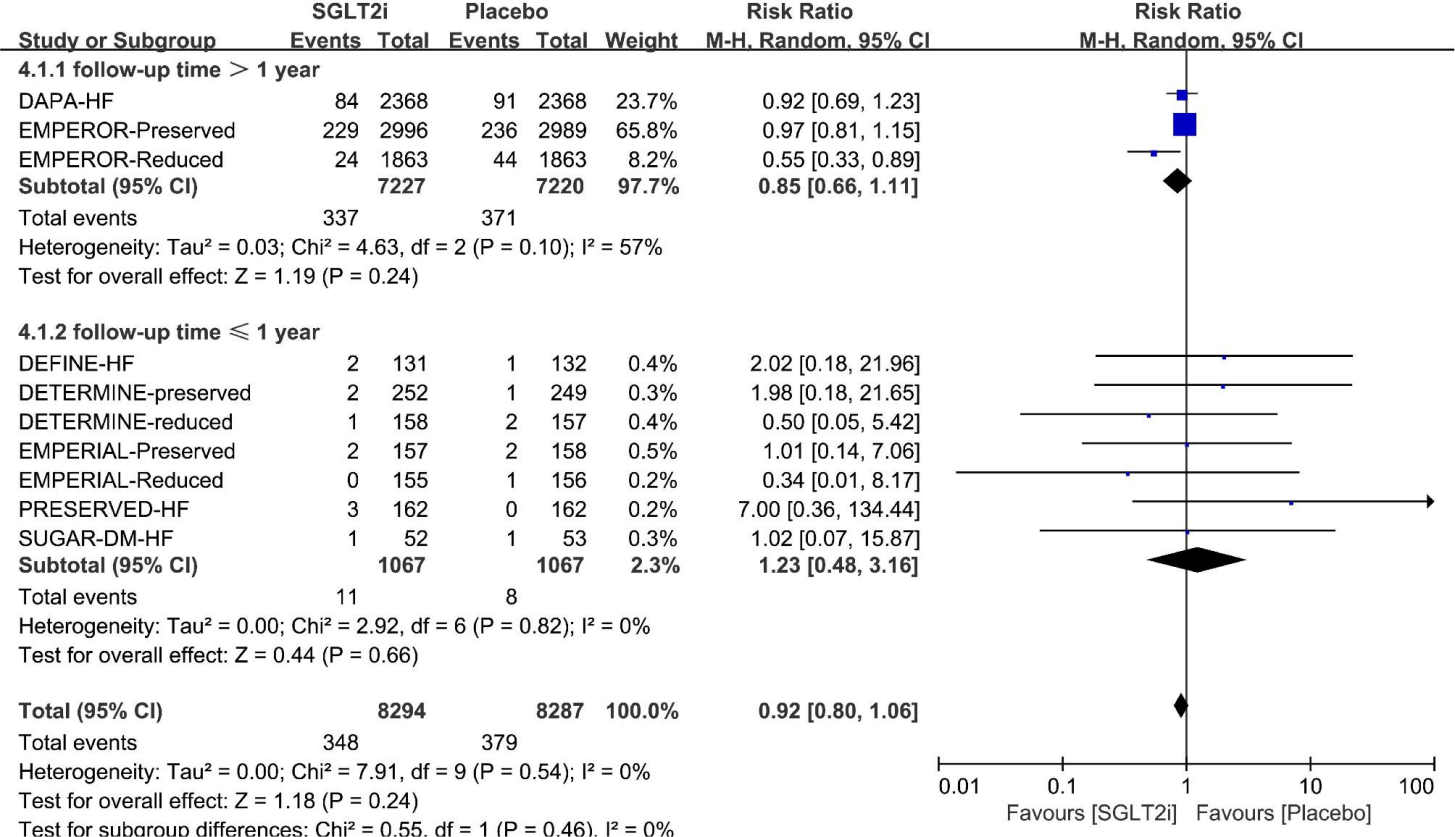
Forest plot of subgroup analysis by follow-up time. M-H = Mantel-Haenszel; SGLT2i = sodium-glucose cotransporter 2 inhibitor

Given that there were three RCTs [9, 31, 32] with duration of follow-up more than 1 years, and the remaining seven RCTs [25–30, 33] with follow-up time less than 1 year, subgroup analysis based on the follow-up duration was also conducted. Since the group with a follow-up duration of more than 1 year displayed moderate heterogeneity (*p* = 0.10, I^2^ = 57%; Fig. 5), the two group were pooled using a random-effect model instead of a fixed-effect model. Our analysis showed no significant difference in the subgroups, regardless of the duration of follow-up (≤ 1 year: RR 1.23, 95% CI 0.48–3.16, p = 0.66; >1 year: RR 0.85, 95% CI 0.66–1.11, *p* = 0.24) (Fig. 5). Publication bias did not exist through Harbord test for either subgroup (≤ 1 year: *p* = 0.28; >1 year: *p* = 0.27, Supplemental Table 2). The result remained consistent after removing studies in sequence (Supplemental Fig. 2c).

**Fig. 6:**
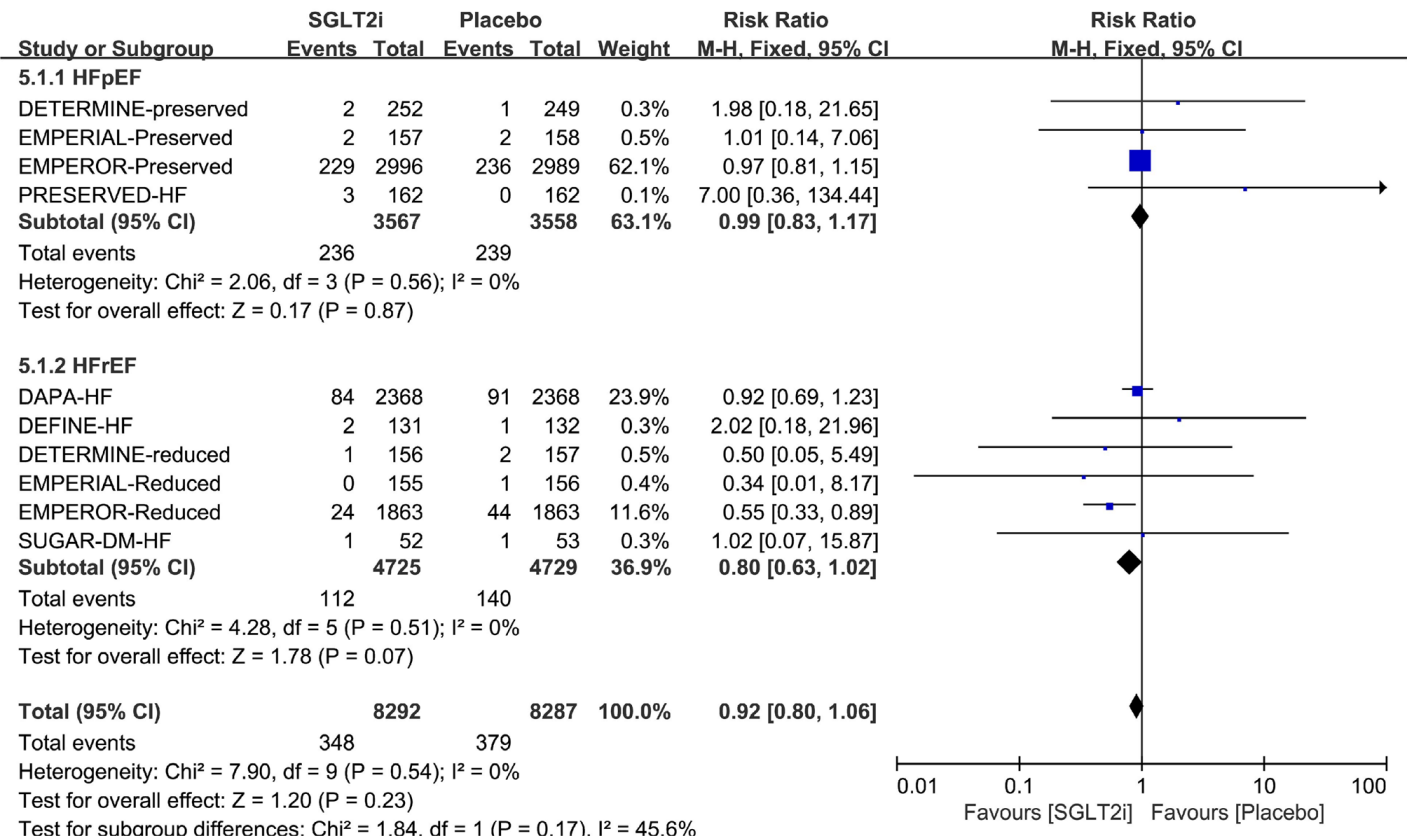
Forest plot of subgroup analysis by type of HF. HF = heart failure; M-H = Mantel-Haenszel; SGLT2i = sodium-glucose cotransporter 2 inhibitor

We also focused on the subgroup analysis by the type of HF, which mainly divided into HFrEF [9, 25, 28, 29, 31, 33] and HFpEF [26, 27, 30, 32] based on ejection fraction. There was no significant heterogeneity across trials (HFpEF: *p* = 0.56, I^2^ = 0%; HFrEF: *p* = 0.51, I^2^ = 0%). Harbord test also did not show any publication bias (HFpEF: *p* = 0.26; HFrEF: p = 0.66; Supplemental Table 2). No significant difference in the risk of AF was observed in any type of HF (HFpEF: RR 0.99, 95% CI 0.83–1.17, *p* = 0.87; HFrEF: RR 0.80, 95% CI 0.63–1.02, *p* = 0.07) (Fig. 6). The funnel plot for assessing risk of bias appeared to be symmetrical in all of the subgroup analyses (Fig. 3).

## Discussion

In this meta-analysis involving 10 RCTS, a total of 16,579 patients with HF were eventually included and 727 AF events were finally identified. The meta-analysis revealed that the use of SGLT2i did not significantly reduce the incidence of AF events in patients with HF, when compared with placebo. What’s more, subgroup analysis based on the type of SGLT2i use, the duration of follow-up, and the type of HF did not yield any significant differences in the AF outcomes. Most of the previous meta-analysis on the relationship between SGLT2i and AF have focused on patients with diabetes mellitus and chronic kidney disease [34, 35]. In contrast, our meta-analysis pay attention to patients with HF. To the best of our knowledge, this is the largest meta-analysis that has investigated the association between SGLT2i use and AF events in patients with HFrEF or HFpEF.

The occurrence and persistence of AF require functional changes that result from disturbed ionic fluxes and altered electrophysiology of the cardiomyocyte [36]. The insufficient cellular energy and oxidative stress caused by mitochondrial dysfunction might contribute to electrical instability and electrical remodeling of AF [37, 38].

The endpoint of our study was the incidence of AF, which has been proved to strongly associated with an increased risk of stroke, hospitalization, and mortality [39–41]. Therefore, preventing the occurrence of AF is crucial. As it is well established, SGLT2i plays a vital role in the management of diabetes and HF, and accumulated evidence suggests their potential in preventing AF [9, 42–45]. Moreover, researchers have demonstrated that SGLT2i could lower the incidence of AF in diabetic patients [46]. Still, the exact mechanism underlying SGLT2i’s ability to reduce the occurrence of AF remains unknown. Nonetheless, some studies have suggested potential mechanisms, such as improvements in mitochondrial function through reduced oxidative stress response, elevated mitochondrial respiration, and increased ATP content [47] as well as the prevention of myocardial fibrosis and hypertrophy [48–50]. These findings suggest that SGLT2i may have a positive impact on reducing the incidence of AF. For instance, both Sfairopoulos and Yin’ studies [20, 51] have reported that SGLT2i therapy was significantly associated with a reduced risk of incident AF in patients with HF, which seemed to be more consistent with the supposed pathophysiological changes. However, unlike Sfairopoulos’s [20] and Yin’s [51] studies, we considered both AF events reported in serious adverse event and other (not including serious) adverse event as primary outcome. Surprisingly, our results led to the opposite conclusion. Our finding in line with a previous meta-analysis, which suggested that there was no significant association between SGLT2i treatment and AF (OR 0.61, 95% CI 0.31–1.19)[52]. As the number of participants (16,579 vs 9454 vs 10,244) and events (727 vs 142 vs 148) are much larger in our meta-analysis, the association we uncovered that there is no significant reduction in the risk of AF with SGLT2i treatment in HF, could be deemed more reliable.

Since all of the AF events were detected by ECG during the follow-up period, it is possible that some paroxysmal AF cases were not recorded, might leading to inaccurate results. Additionally, the short mean follow-up time might contribute to undetected differences between SGLT2i and placebo in our analysis. Among all trails included, the heaviest weight of the statistical analysis depended on EMPEROR-preserved, where empagliflozin proved to be more effective in reducing the risk of exacerbation of HF than dapagliflozin [53]. Furthermore, the empagliflozin group had more significant beneficial effects on high-density lipoprotein (HDL) and low-density lipoprotein (LDL) and lower glycated hemoglobin levels than dapagliflozin [54, 55]. Interestingly, we found that empagliflozin present an effective role in reducing the risk of AF after removing EMPEROR-preserved in the sensitivity analysis. Since diabetes and CVD are well-established risk factors for AF and cardiac arrhythmias [56, 57], which might explain the different results seen in the sensitivity analysis between dapagliflozin and empagliflozin studies. Nevertheless, the relation between SGLT2i and AF is much more to explore. Further RCTs that explicitly define the AF outcomes are needed to confirm the association reported in the current study.

There are several limitations to this analysis. First, to the best of our knowledge, none of the trials put AF as the primary endpoint event, which may lead to our conclusions being inconsistent with reality. Additionally, trials could not be grouped according to the comorbidities because patient-level data were not available and not all trials reported baseline prevalence of diabetes, chronic kidney disease, coronary heart disease. Further studies are warranted to verify and expand on these findings. Second, DELIVER [58], a novel registered trail about dapagliflozin, was excluded since we couldn’t find the data we were interested in. Third, because all trials used the same concentration of SGLT2i, the trials could not be grouped by dose. Fourthly, the weight ratio of some RCTs was so huge that it may cause the bias risk of the results after sensitivity analysis. Fifth, the AF events recorded did not differentiate between persistent AF and paroxysmal AF. The latter is difficult to detect unless an explicit AF episode occurs during the exploration, which makes our results less robust. Lastly, it is noteworthy that traditional anti-HF drugs have been reported to lower the incidence of AF. Nevertheless, due to the lack of data regarding the number of events linked to anti-HF drugs used, we should exercise caution in interpreting the results of subgroup analyses. In conclusion, Future trials with AF as the primary outcome make sense.

In summary, this analysis suggests that SGLT2i may not prevent the occurrence of AF in patients with HF. Therefore, more studies should be conducted in patients with HF to demonstrate the effects of SGLT2i on AF.

## Electronic supplementary material

Below is the link to the electronic supplementary material.

**Supplemental Figure 1**. Summary of risk of bias across all included studies. **Supplemental Figure 2**. Removing studies one by one in sensitivity analysis (a) Removing studies one by one in sensitivity analyse for all trails; (b) trails used empagliflozin; (c) trails follow-up time > 1 year. **Supplemental Figure 3**. Removing EMPEROR-Preserved in empagliflozin group M-H = Mantel-Haenszel; CI = confidence interval. **Supplemental Table 1**. PRISMA checklist. **Supplemental Table 2**. Harbord test

## Data Availability

The datasets generated and/or analysed during the current study are available in the ClinicalTrials.gov database, https://clinicaltrials.gov/.

## Abbreviations

AF: Atrial Fibrillation
CI: Confidence Interval
HF: Heart Failure
HFrEF: Heart Failure with Reduced Ejection Fraction
HFpEF: Heart Failure with Preserved Ejection Fraction
LVEF: Left Ventricular Ejection Fraction
NOAF: New-onset Atrial Fibrillation
RCTs: Randiomized Controlled Trails
RR: Risk Ratio
SGLT2i: Sodium-Glucose Cotransporter 2 Inhibitor
